# Institutional Organisation in America

**Published:** 1914-07-18

**Authors:** 


### INSTITUTIONAL ORGANISATION IN AMERICA.

### II. A Lesson from the Massachusetts General Hospital, Boston.*

In a previous article we discussed the attempts that have been made in New York to set up a criterion by which out-patient clinics for children may be classified. In one or two respects, as was pointed out, the management of American institutions has already diverged from the general principles upon which hospital evolution has proceeded in this country, as, for instance, in the matter of medical discipline and control. In Britain out-patients are treated, in the great majority of cases, by the junior members of the visiting staff; and this is true both of children's and other special departments, as well as of the adult clinics. As our readers are well aware, each of these out-patient or assistant physicians and surgeons is responsible only to his own conscience and to the managers of the hospital; he is not in any way controlled by or subject to the senior staff or any of his colleagues whatever. Indeed he can, and, on occasions which are not so very rare, does adopt methods of treatment or take other professional actions of which some or all his colleagues may disapprove; and they cannot call him to account in any way. In America this organisation is being steadily departed from. In many of the most famous modern institutions there, a system somewhat resembling the chain of command in military service has been established. Responsible to the governing body there is a head physician or surgeon, whose title may be director, chief of clinic, or what not. Working under the orders of this directing authority are a small band of trusted experts, often called associate physicians or surgeons. Each of these in his turn commands a band of more junior doctors, corresponding more or less to the assistant staff in this country, under whom in turn are residents of varying degrees of seniority and authority.

### THE BOSTON ORGANISATION.

In the Massachusetts General Hospital Dr. Fritz B. Talbot has been for the last four years the chief of service of the children's medical department; and the perfection of the American system to which he has brought his out-patient clinic has excited the admiration even of New York. Dr. Richard M. Smith describes† the organisation of this clinic, which is certainly most instructive and extraordinarily complete. We doubt whether any British out-patient department can show arrangements as thorough and methodical for the care (and after-care) of the patients. The plan adopted must necessarily be very expensive. To that extent it may at the moment be impracticable to emulate it in its entirety in this country; but there are many clinics which might well bestir themselves into efforts to approach nearer to the example of efficiency set by the Boston department.

Dr. Smith begins with a dogmatic statement which has obtained the approval of the best hospital administrators in New York, as noted in our previous article; in our judgment this dogma contains a very important truth, too often overlooked or neglected in Great Britain. He says: "Frequently one physician has to see so many patients that he has no time for the thorough examination or study of any single individual. If one man sees only new patients every day he has no opportunity to follow the subsequent course of these patients. There should be enough men on duty at the same time so that no man lacks time for careful consideration of each patient. Every physician on duty should have at least certain days when he is not compelled to see new patients, but may devote himself to patients whom he has seen previously and wishes to follow."

It is evident that to secure such order in an out-patient clinic, and to ensure its maintenance, some supervision is needed, closer and more continuous than any board of governors can supply. In other words, a medical chief of clinic, wielding authority over his colleagues, is necessary. And this is, in fact, the keynote of the system. The chief of clinic has power to formulate and carry out a definite line of policy, to which all his subordinates must conform; and to change it, or experiment with it, as he thinks fit and when he thinks fit. Otherwise, as Dr. Smith says, the number of men in charge during the year is so great that the method of running the clinic and the treatment of the patients lack consistency and uniformity.

In the Massachusetts General Hospital children's department two men are on duty in the out-patient clinic at the same time, and each one sees the new patients on alternate days. The man who sees new patients on Monday, Wednesday, and Friday tells these patients to return on a Tuesday, Thursday, or Saturday, when he is free to see them. This adds much to the value of the clinic for the physician, and establishes a personal relation between physician and patient which is beneficial to both. If the patient presents no condition which requires special study, he is allowed to return on any day that is convenient to himself and is seen by the junior house officer. If he presents a condition which is being specially studied by some other physician in the clinic, he is seen by that doctor on subsequent visits. It will be noticed that the out-patient medical officers are expected to attend every day of the week—a demand upon their time and energy which few British hospitals could ask and few physicians or surgeons could supply.

The treatment of many patients, in fact of most patients, in a children's clinic demands a knowledge and appreciation of the home conditions and environment. This contribution can be made only by a social worker. Indeed, Dr. Smith says that the time is past when it is possible to conduct an out-patient clinic without such officers, who must be trained especially for their positions and be

* Article I. was published in THE HOSPITAL of July 11, 1914.

† *Archives of Pediatrics,* May 1914, p. 350.

thoroughly familiar with the problems of infancy and childhood. They should be closely connected with the clinic, so that there shall be no separation of their work and the physician's. The results of their study should constitute a part of the patient's record. At Boston it has been found advisable to have a worker for infants and one for children, with assistants and volunteers working under them. This system is not put forward as the only possible or legitimate one; it is admitted that some other might suit other conditions and other clinics better.

Once a week a conference is held between members of the medical staff and the social workers. Matters of general interest with reference to the clinic are discussed and particular cases presented. The medical history of each case is given by the physician who has been following it, and the social history is given by the worker. Then the general problem or the special difficulty which that case presents is discussed by the whole group. This conference serves as a means of keeping all members of the department informed about what is taking place in the clinic, and also gives each patient the benefit of the combined advice of the whole staff of physicians and social workers. These conferences have proved, it is stated, of distinct benefit. We feel sure their establishment, or rather their successful maintenance, would not be easy in this country; for the tendency to perfunctoriness must be very difficult to keep at bay, even when there is an exceptionally gifted chief of clinic to sustain the enthusiasm and ensure the affectionate regard of his junior officers. The system is Teutonic rather than British in its appeal; Englishmen are not good at this sort of work, and possibly it is the large German element in the American medical profession which has contributed to its success.

Another labour which is quite foreign to British methods is the "clinic survey." When one of these is decided upon, the records of all patients seen during a certain period of time are studied, and certain forms are filled up with details of the number of attendances, results of treatment, and information about many other points, medical and sociological. The diagnoses filled in on the forms enable the chief of clinic to analyse his patients and to classify them. Thus one large group consists of normal children who have required merely regulation of their diet to correct the indiscretions of their guardians. Another group is that of children with cardiac lesions, and so on with other conditions. The survey shows also what percentage of patients attend once only, a rough and ready test in inverse proportion of their appreciation of the medical service. It shows also the percentage of cases submitted to clinical laboratory methods, the results of treatment, and other points.

The inevitable tendency to specialisation in modern medicine has long been noted and is steadily progressing everywhere. This tendency is probably accelerated by the exact division of labour brought about by the Boston system. Thus we learn that as a result of the "clinic survey" cases are divided into groups, each of which is allotted to one physician and to one social worker. In the cardiac group, for instance, one physician sees the patients on a given day throughout the year, thus ensuring continuous and uniform treatment. Furthermore, the relation of any particular patient and the disease which he has to that disease as it affects the community is presented by this group study in a way which would be impossible from a consideration of isolated cases. There is an intimate connection between out-patient work and the work done by various public health and relief agencies in the city. Co-operation with these agencies in dealing with the individual child and in the study of preventive medicine is strongly advocated by Dr. Smith. This argument is one which will find ready sympathy among British institutional workers. It may be true that the principle has been carried further in selected American cities —Boston, for example—than with us; but at least the principle is recognised and admitted amongst us as desirable and inevitable.

The primary classification of the actual children, as carried out in the Massachusetts General Hospital, is into three main groups—the health cases, the chronic cases, and the acute cases.

The health clinic cases are seen in a separate room, so as not to expose the patients to infection from those acutely ill. The largest sub-group in this class is naturally that of infant feeding. Many of the babies seen here are referred at once to the milk station for their future treatment. Breast feeding is encouraged to the utmost; if it is not feasible, instruction in the home modification of cow's milk is regarded as the first substitute. Modifications prepared in the laboratory are, of course, often used, but not unless the other plans fail. There is an arrangement with a milk station to secure certified milk (either unmodified or modified) at a price adapted to the means of the family. Each case is investigated by the social worker, and the price is fixed by her. After the patients have been to the hospital, they are visited at home and taught how to do the things which have been told them in the clinic.

In the chronic group, rickets is chosen in Dr. Smith's paper as a case in point. In the treatment of this disease the children's department works in close co-operation with the orthopædic department. The children's department directs the general medical care, together with the home visiting and the instruction in hygiene. The orthopædic department seeks to prevent deformities, or to treat them when they arise. Moreover, the children's department charges itself with the oversight of the other members of the family, to prevent, if possible, the development of rickets in the younger members.

Acute cases are similarly subdivided and specialised. All cases of endocarditis, chorea, and rheumatism are considered together; this large group is selected merely as typical of the working of the organisation. After the need of active treatment has passed away, the child is kept under continuous supervision so as to ensure as far as practicable against future breakdown. On return to school the school nurse and the teacher are communicated with so as to provide that the child is assigned to a ground-floor class and watched to prevent over-exertion. During the period of enforced quiet at home, before return to school can be contemplated, amusement of some sort is provided, adapted to the individual child. Thus there is available a loan collection of games and occupations, and helpers are enlisted who will read to or give lessons to those who are well enough.

All these thorough-going methods mean a great expenditure of labour and forethought. To keep this side of the clinic's activities in working order a (non-medical) "clinic manager" is appointed, whose sole duty is to supervise the running of the clinic. This officer at the Massachusetts General Hospital is a lady. She sees that patients are examined as promptly as possible and by the right physician; that they go promptly to other departments if referred thither; that they are instructed when to return; that their records are properly kept; and that the follow-up system is running smoothly and regularly.

All this means expense, as has been already remarked. Considerations of expense must not be allowed to intervene if better work can be done by these newer methods, so thorough and elaborate in their organisation. If there is any lesson which has been more fully learnt and taken to heart by the wisest institutional administrators in this country as a result of their experience of the voluntary system, it is that efficiency is in the long run the surest guarantee of liberal public support; and that even costly schemes are really paying propositions, provided they do clearly and definitely mean improved service for the hospital patients and provided this can be brought home to the benevolent classes on whom the hospitals rely for their income. The scheme of organisation adopted in Boston must be of great interest to British hospital administrators, and there is much in it which, we feel sure, will commend itself to them, either for imitation or adoption.

